# An Open-Circuit Indirect Calorimetry Head Hood System for Measuring Methane Emission and Energy Metabolism in Small Ruminants

**DOI:** 10.3390/ani9060380

**Published:** 2019-06-21

**Authors:** Carlos Fernández, Julio Gomis-Tena, Alberto Hernández, Javier Saiz

**Affiliations:** 1 Departamento de Ciencia Animal, Universitat Politècnica de Valencia, 46022 Valencia, Spain; 2 Centro de Investigación e Innovación en Bioingeniería, Universitat Politècnica de Valencia, 46022 Valencia, Spain; jgomiste@eln.upv.es (J.G.-T.); ahernanf@eln.upv.es (A.H.); jsaiz@ci2b.upv.es (J.S.)

**Keywords:** goats, indirect calorimetry, data acquisition

## Abstract

**Simple Summary:**

An open-circuit indirect calorimetry system for small ruminants was updated. Calibration factors for CH_4_, CO_2_ and O_2_ close to 1 confirmed the absence of leaks in the indirect calorimetry system and the accurate performance of this device. An experimental test quantified the gas exchange and the repeatability for CH_4_ and heat production measurements, which were 79% and 61%, respectively. The heat production obtained by indirect calorimetry was close to the heat production obtained by carbon and nitrogen balance. Discrepancies between the two methods averaged 1.92% when expressed as a percentage of the intake of metabolizable energy, a rather satisfactory value considering the substantial amount of technical and analytical work involved. The close agreement found between them can be considered as an indicative of the absence of systematic error. When diets with different forages were compared, the daily CH_4_ production was 1.54 and 1.25 L/h for diets-based in alfalfa hay and alfalfa silage, respectively.

**Abstract:**

Methane (CH_4_) is a natural by-product of microbial fermentation in the rumen and is a powerful greenhouse gas. An open-circuit indirect calorimetry system for continuous determination of CH_4_ and CO_2_ production and O_2_ consumption and, thereafter, heat production (HP) calculation for small ruminants was described and validated. The system consisted of a computerized control, data acquisition and recording system for gases and air flux. The average value ± standard deviation for the calibration factors in the system were 1.005 ± 0.0007 (n = 6), 1.013 ± 0.0012 (n = 6) and 0.988 ± 0.0035 (n = 6) for O_2_, CO_2_ and CH_4_, respectively. Calibration factors close to 1 confirmed the absence of leaks in the indirect calorimetry system. In addition, an experimental test with 8 goats at mid lactation was conducted to validate the system. The repeatability for CH_4_ and heat production measured with the open-circuit indirect calorimetry system was 79% and 61%, respectively. Daily average HP measured by indirect calorimetry (Respiration Quotient method) was close to the average HP determined from Carbon-Nitrogen balance (CN method), accounting for 685 and 667 kJ per kg metabolic body weight, respectively. Therefore, discrepancies averaged 1.92%, a rather satisfactory value considering the substantial amount of technical and analytical work involved. The close agreement found between both methods can be considered as being indicative of the absence of systematic error. Two diets with different forage were tested: 40% was either alfalfa hay (HAY) or alfalfa silage (SIL), and the proportion of concentrate was the same in both groups (60%). The experimental trial shown that HP and CH_4_ were higher in HAY than SIL diet (differences between treatments of 28 kJ of HP per kg of metabolic body weight and 7.1 L CH_4_/day were found). The data acquisition and recording device developed improved the accuracy of the indirect calorimetry system by reducing the work involved in managing output data and refining the functionality for measuring gas exchange and energy metabolism in small ruminants.

## 1. Introduction

Agreement was reached at the 2015 United Nations Climate Change Conference in Paris to keep global warning below 2 °C. In addition to reductions in CO_2_ emissions, substantial reductions in short-lived climate pollutants such as methane (CH_4_) will be needed to achieve the target. The CH_4_ conversion factor (Ym) was introduced by the Intergovernmental Panel on Climate Change (IPCC) to indicate the proportion of an animal’s gross energy that is converted to enteric CH_4_ energy, and it is widely used for national greenhouse gases (GHG) inventories and global research on mitigation strategies. However, it has been consistently shown that CH_4_ emissions are related not only to feed intake but also to feed composition [1]. The estimates, following the International Panel of Climate Change guidelines, for national GHG inventories are generally developed based on equations for cattle, and had limited applicability for other ruminants and camelids. Therefore, specific data for CH_4_ emission from other ruminants and camelids, which have different metabolic rate than cattle [2], are necessary for calculation of GHG budgets of countries.

Direct measurement of enteric CH_4_ emissions from ruminants requires specialized equipment [3]. Calorimetry devices have been built, in part, to measure emissions and assess the mitigating effects of dietary manipulations. The principle behind open-circuit indirect calorimetry techniques is that outside air is circulating around the animal´s head, mouth and nose, and the expired air is collected [4,5]. Gaseous exchange is then determined by measuring the total airflow through the system and the difference in gas concentrations between inspired and expired air. Open-circuit indirect calorimetry systems are, therefore, an indirect calorimetry method that consists of measuring the gas exchange associated with the oxidation of energy substrates and determining the associated heat production (HP). Quantitative measurements of gas exchange in respiration units have been used in indirect calorimetry to estimate the HP in animals.

Usually, indirect calorimeters are associated with high cost facilities where respirometry chambers and equipment are installed in a laboratory building [4]. When the cost of building facilities is prohibitive, alternative less expensive systems have been investigated. These may be more readily used in a production setting. One option is to build an open-circuit indirect calorimetry system based on a facemask [6] or head hood boxes [7], which can quantify gaseous exchange using similar principles. Since CH_4_ and CO_2_ emission is closely related to feed intake [5] and the facemask prevents the animal from eating during measurement, the use of head hood boxes is preferred for long term measurements [7]. This also improves the accuracy of measurement compared to the facemask method.

The objective of this study was to update the open-circuit indirect calorimetry system described by Fernández et al. [6] with two head hoods and a data acquisition unit. A technical test evaluated the open-circuit indirect calorimetry system and an experimental test was used as external evaluation of the respirometry system. Heat production determined by indirect calorimetry (respiratory quotient; RQ method) was also compared with the carbon-nitrogen balance (CN method).

## 2. Materials and Methods

### 2.1. Ethics Statement

The experimental procedure used by the Animal Use and Care Committee of the Polytechnic University of Valencia (Spain) was approved (2017/VSC/PEA/00182). The codes of practice for animals used in experimental work proposed by the Spanish guidelines for experimental animal protection [8] were followed. In addition, the Animal Science Department from the Polytechnic University of Valencia provided veterinary researchers that ensured that goat management followed the codes of practice for animals used in experimental works proposed in the European Union [8]. The authors declare that this manuscript does not involve ethical issues or affect any endangered or protected species.

### 2.2. Open-Circuit Respiration System

**Figure 1:**
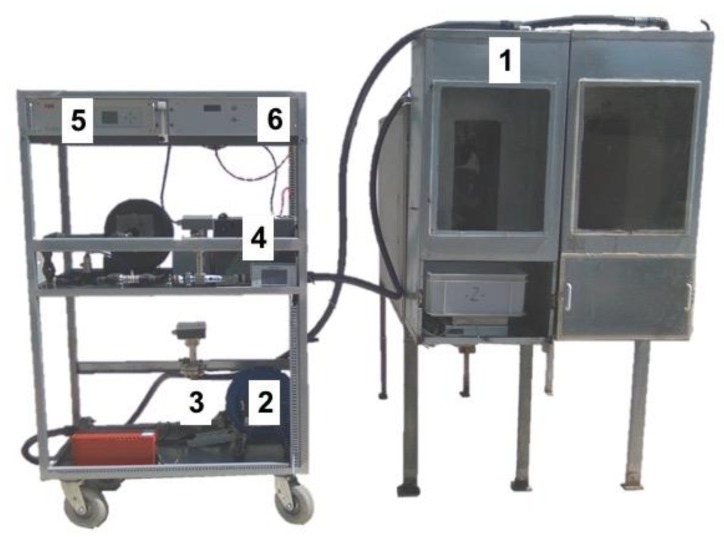
Mobile open-circuit indirect calorimetry equipment cart. (1) Head hood, (2) fan, (3) mass flowmeter, (4) gas cooler, (5) gas analyzer (oxygen, carbon dioxide and methane), (6) box for system control and data acquisition panel.

The open-circuit indirect calorimetry system for measuring real time gaseous exchange in small ruminants (sheep and goats) using a facemask was described by [6]. Based on this system design, a new respirometry unit with two head hood boxes connected to one analyzer was built and developed. The instrumentation was installed on a mobile cart to make the system portable to any location with electricity supply (Figure 1). 

Each head hood was made with 1.5 mm galvanized plate (530 mm long × 1160 mm high × 360 mm wide; volume = 219 liters). It was suspended on the front of the metabolic cage by two hooks placed on its rear side. The hood had a transparent acrylic (bolted and glued with silicon on the edge) window at the front (420 mm long × 530 mm high), and a drawer (500 mm long × 290 mm high × 350 mm wide) to allow food and water to be added to a bucket. Two lateral locks situated in its front side and main body of the head hood locked the drawer. Atmospheric air entered into the head hood through an orifice (internal diameter 20 mm) made on its top on the opposite side to the main line that draws air out of the hood. 

**Figure 2:**
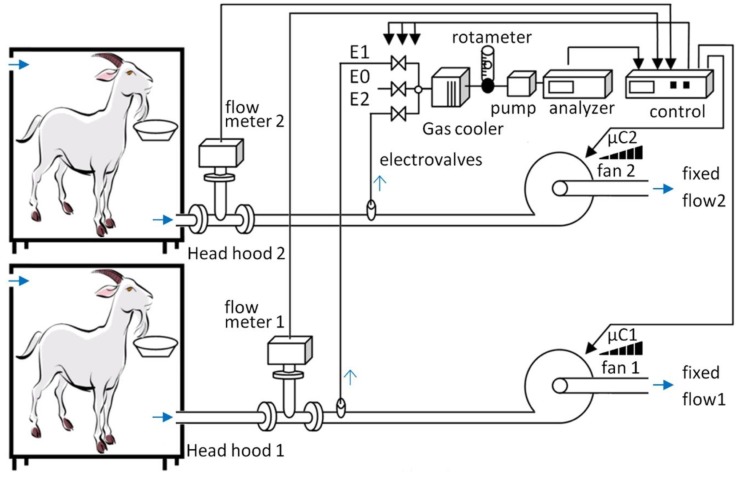
Schematic design of the mobile calorimetry system.

The indirect calorimetry unit was based on two separate but linked sampling lines, each associate with one of the two head hoods (Figure 2) mounted on a cart on wheels (106 cm long by 173 cm high by 5 cm wide; Figure 1). Each main line drew air across the head hood through a PVC tube (id 25 mm), equipped with an air filter to reduce dust in the hood and connected to a centrifugal fan (CST60; Soler Palau Inc., Parets del Vallès, Barcelona, Spain) that expelled gas to the outside of the head hood and provided air flow through the system. Total air flow was measured by a mass flowmeter with a range from 0 to 10,000 L/h (Thermal Mass Flowmeter Sensyflow VT-S; ABB Automation Products GmbH, Alzenau, Germany). A secondary line (internal diameter 5 mm) situated after the mass flowmeter, took a gas sample from the main line though to membrane pump (ABB Automation Products GmbH, Alzenau, Germany) to the rotameter (DK800; ABB, Alzenau, Germany). The gas sample then passed through the gas cooler (SCC-C; ABB Automation Products GmbH, Alzenau, Germany) to remove moisture prior to its entry into the gas analyzer unit.

The upper part of the cart accommodates the gas analyzer unit (Easyline Continuous Gas Analyzer, model EL 3020; ABB Automation Products GmbH, Alzenau, Germany) and the data acquisition device for system control. The gas analyzer measured three gases (CH_4_, CO_2_ and O_2_) in parallel with the data acquisition device (see Figure 1 and Figure 2). The CH_4_ and CO_2_ are measured using infrared principle with a range from 0–0.15 and 0–1.5%, respectively. The analysis of O_2_ works on the paramagnetic principle with a range from 18–21%. The paramagnetic O_2_ analyzer was equipped with an atmospheric compensation module to account for changes in atmospheric pressure. To analyze the gas from the two head hoods, a switch using electrovalves was connected to the data acquisition device (see calculation section for detail of the sequence and its length).

### 2.3. Data Acquisition

The data acquisition and recording system was completely made and designed by the Centro de Investigación e Innovación en Bioingeniería from the Universitat Politècnica de Valencia (Valencia, Spain; Figure 1: box label with the number 6). 

The calorimetry recording system was controlled by an electronic circuit based on three dsPIC33FJ128MC804 microcontrollers (Microchip Technology Inc., Chandler, Arizona, USA). Two of them were dedicated to airflow control (one for each line). Each airflow meter was monitored by a 12-bit analogue-to-digital converter included in the microcontroller. To keep the airflow fixed, microcontrollers coordinated the speed of the fans via transistors (FQA140N10; Fairchild; On Semiconductor, Phoenix, USA) and diodes (V80170PW-M3/4W; Vishay InterTechnology, Inc., Malvern, PA, USA). The third microcontroller executes 6 tasks: (1) reading the configuration parameters from a text file of a microSD memory card (SanDisk 2 GB microSD Memory Card, Western Digital Corp., California, USA), (2) interaction with the user, (3) constant airflow controller (4) activation of electrovalves, (5) recording measurement of gas from analyzer, (6) storing the results in a text file on the same microSD memory card.

Asynchronous serial communication between the microcontrollers (proprietary protocol) and between microcontroller and the gas analyzer (Modbus Organization Inc., Hopkinton, MA, USA) was used. A deliberately minimalistic user interface was chosen consisting of 2 buttons: one for data acquisition start and one for acquisition stop, and a 4 × 20 character Liquid Cristal Display showing the measurement values.

### 2.4. Technical Test

#### 2.4.1. Analyzer Calibration

The analyzers were calibrated with gases of known concentrations (81%N_2_:19%O_2_ and 77.35%N_2_: 21%O_2_:1.5%CO_2_:0.15%CH_4_). Calibration of the analyzers involved the following steps; (1) initial zero calibration using N_2_ gas, (2) use of known mix of gasses as upper limit, and (3) final calibration of the unit with atmospheric air.

#### 2.4.2. Whole System Calibration

Besides adjusting and calibrating the analyzers, it was also necessary to check the whole system efficiency for gasses detection by simulating the gas exchange produced by an animal inside the head hood. Therefore, the whole system was calibrated by injecting pure gas N_2_ (0.9999), CO_2_ (0.9999) and CH_4_ (0.9999) into the head hood [4] to produce an O_2_ decrement and a CO_2_ and CH_4_ increment. Calibration factors comparing the volume of gas injected and detected by the system were obtained. Total gas released was determined gravimetrically using a precision electronic scale (MOBBA mini-SP 0.2–30 kg) (Mobba Industrial Catalunya S.A., 08916 Barcelona, España). Sufficient gas was released (about 380, 160 and 75 g of N_2_, CO_2_ and CH_4_ respectively, for 6 h) to give acceptable accuracy in the measurement of the change in cylinder weights and injected at the required flow rate to simulate the O_2_, CO_2_ and CH_4_ exchange produced by an animal in the system. Calibration factors were calculated according to Brockway et al. [9]. 

### 2.5. Calculations

Methane and CO_2_ production and O_2_ consumption were calculated as described previously [10] using the Haldane transformation, except that no theoretical values for atmospheric CH_4_, CO_2_ and O_2_ concentrations were used, because the thermal mass flowmeter corrected for standard temperature and pressure. 

Before gas measurement, atmospheric air was sampled. To set the electrovalve activation sequence and duration that were programmed in the third microcontroller, we determined a steady-state establishment interval value. We switched the analyzer input between two well-known composition gas mixtures and obtained the correct value at 22 s. Consequently, the switching intervals were set to a value greater than 22 s (30 s). The end of that interval indicated that the analyzer was correctly calibrated, and the measurement was saved.

To obtain the background atmospheric gas levels used in the calculations these were called CONTROL. The selected sequence was: (30 s) CONTROL, (30 s) line 1 (head hood 1), (30 s) CONTROL and (30 s) line 2 (head hood 2). Subsequently, a complete measurement sequence took 2 min and the values corresponding to line 1, line 2 and CONTROL were stored on the memory card every 2 min.

### 2.6. Experimental Test

#### 2.6.1. Animals and Feeding

To validate the system, experimental energy balance data was obtained with 8 goats. Eight Murciano-Granadina dairy goats at mid lactation (16 weeks), with similar body weight (BW; 39 ± 1.1 kg) were selected to determine gas exchange. Each goat was offered once daily 2.5 kg of feed per day, comprising 1.0 kg forage and 1.5 kg of a standard concentrate. The two diets (Table 1) were composed of 40% forage (either alfalfa hay [HAY] or alfalfa silage [SIL]) and 60% of concentrate, following the recommendations of Agricultural and Food Research Council (AFRC) [11] and Fundación Española para el Desarrollo de la Nutrición Animal (FEDNA) [12]. Four goats received each diet. All goats were housed in a building in which the environment was controlled by a HOBO device (HOBO probe, Onset Data Loggers, Cape Cod, MA, USA) at thermo-neutrality (20–23 °C). 

#### 2.6.2. Energy and CN Balances, and Heat Production (HP) Calculation

The goats were kept in individual floor pens for a period of 15 days to adapt to their allocated experimental diets. Then goats were moved to individual metabolism crates for 10 days. Feed intake, total fecal and urine output, and milk were recorded daily for each goat over a 5-day period. Representative samples of diet, feces, urine and milk were collected daily, stored at −20 °C, and pooled for chemical analysis.

After collecting samples for determining energy and CN balances, gaseous emissions from each goat were measured for a period of 22 h by housing them in individual metabolism crates fitted with the respirometry units (2 animals per day for 4 days). Each goat was weighed before being placed in the metabolism crate and was given access to its daily feed allocation at 0900 h. Individual daily outputs of urine were collected into buckets containing 100 mL of 10% sulphuric acid (H_2_SO_4_), and feed intakes were recorded by measuring the difference between feeds offered and refused. Before being removed from the metabolism crates, each goat was milked at 0800 h with a portable milking machine (Flaco, model DL-170, J. Delgado S.A., Ciudad Real, Spain). 

The equation developed by [13] for calculation of HP based on the respiratory quotient from measurements of gas exchange (O_2_ consumption, CO_2_ and CH_4_ production) and nitrogen excretion in urine (N_urine_) was used:

$$HP\;\left(kJ\right)=\;16.18\;\times \;O_{2}+5.02\times CO_{2}-2.17\times CH_{4}-5.99\;\times \;N_{urine}$$

where gases were expressed in liters per day and N_urine_ in g/day.

Respiratory quotient (RQ) was calculated as CO_2_ produced: O_2_ consumed ratio. The metabolizable energy (ME) intake (MEI) was calculated as the difference between gross energy (GE) intake and energy losses in feces, urine and CH_4_ (with an energy equivalent value of 39.54 kJ/L; [14]). Retained energy (RE) was determined as the difference between MEI and HP. Retained energy determined with the CN method was calculated according to [13] from the C (g) and N (g) balance (RE = 51.8 × C − 19.4 × N). Heat production was calculated as difference between MEI and RE.

Feed and feces were dried in a forced air oven at 55 °C for 48 h and then ground to pass 1 mm screen. Urine and milk were dried by lyophilization. Chemical analyses were conducted according to the methods of AOAC [14] for dry matter (no. 934.01), ash (no. 942.05), ether extract (no. 920.39) and crude protein (no. 968.06). Gross energy content was determined in an adiabatic bomb calorimeter (Gallenkamp Autobomb; Loughborough, UK). Acid detergent fiber (ADF) and neutral detergent fiber (NDF) of diets were determined using filter bags and a fiber analyzer (A220; ANKOM Technologies, Fairport, NY, USA) following AOAC [14] official methods (no. 973.18) according to [15]. Acid detergent lignin (ADL) was determined according to Robertson et al. [16]. Carbon and N were analyzed by Dumas principle (TruSpec CN; LECO Corporation, St. Joseph, MI, USA).

### 2.7. Data Analysis

To calibrate the whole system in the technical test, pure gas was injected into the head hood and the resultant response of the system was compared with the quantity of each gas injected, accurately determined by gravimetric approach.

In the experimental trial the repeatability between repeated measurements of the same animal measured with the indirect calorimetry system was estimated as a function of the variance components for animals and the residual variance (library SixSigma from R [17]). Then, the RQ method and the CN method for HP were compared; discrepancy between methods was calculate with next index; [(HP_RQ_ – HP_CN_)/(MEI)] × 100). Finally, the effects of diet and time on goat gas exchange were analysed using the mixed model (lme function from the nlme library) from R [17]. Least square means were reported throughout for the fixed effect of diet and differences were considered significant at *p* < 0.05.

## 3. Results and Discussion

### 3.1. Technical Test

The calibration factors obtained by injecting pure gas from the cylinder into the head hood and the resultant recovery from the system are shown in Table 2. The means of 6 calibration factors and standard deviations for the open-circuit indirect calorimetry system were 1.005 ± 0.0007 (n = 6), 1.013 ± 0.0012 (n = 6) and 0.988 ± 0.0035 (n = 6) for O_2_, CO_2_ and CH_4_, respectively (Table 2). The accuracy of the gas exchange determination is further dependent on the ability of the system to measure gas composition and the total volume of the air moved through the open-circuit indirect calorimetry system [3,4]. We observed that all the calibration factors were very close to 1, demonstrating the absence of leaks and the accurate performance of the indirect calorimetry system.

### 3.2. Experimental Test

Gage Repeatability and Reproducibility (Gage R & R) is a methodology used to define the amount of variation in measurement data in an open-circuit calorimetry system. It compares the measurement variation to the total variability observed, consequently defining the capability of the measurement system. Measurement variation consists of two important factors, repeatability and reproducibility. Repeatability is due to equipment variation (indirect calorimeter device) and reproducibility is due to operator variation (does not apply in our study). The results from the repeatability analysis of CH_4_ and HP are shown in Table 3.

The repeatability for CH_4_ and HP measurements was 79% and 61%, respectively. The study of Robinson et al. [18] reported a repeatability of 79% and 81% for CH_4_ and HP, respectively, and in the study of Robinson et al. [19], also conducted in sheep, a repeatability value for CH_4_ of 76% and 60% for HP was reported. Both studies used portable accumulation chambers. Other studies [20] in sheep with respiration chambers found repeatability values of 89% for CH_4_. Oddy et al. [21] using respiration chambers and sheep in different physiological stages found a repeatability value of 65% for CH_4_. Our repeatability was in the range of values reported in the literature, although an improvement may be achieved when CH_4_ and HP were adjusted for live weight and feed intake [18,19,20,21].

The HP values determined in each goat by the CN method are shown in Table 4. The energy balance measurements were carried out with the aim of evaluating discrepancies in the estimation of HP determined by RQ and the CN methods. The CN method is frequently determined in association with indirect calorimetry measurements [22], and it depends on measurements of C and N intake and their losses as urine, feces and gases (CO_2_ and CH_4_). Therefore, the RQ and the CN methods are not completely independent of each other. The HP determined with the CN method was lower than the calculated with the RQ method. The CN method generally results in an underestimation of HP because CN balance is usually underestimated due to evaporative and other losses in excreta [22,23,24]. The agreement observed in our study between both methods can be another index of the system reliability. Discrepancies averaged 1.92% when expressed as a percentage of the MEI, a rather satisfactory value considering the substantial amount of technical and analytical work involved. The close agreement found between the two methods can be considered as an indicative of the absence of systematic error. Other authors [10], feeding wethers at about maintenance level, and working with respirometry chambers and CN method, obtained discrepancies of 1.84%.

**Figure 3:**
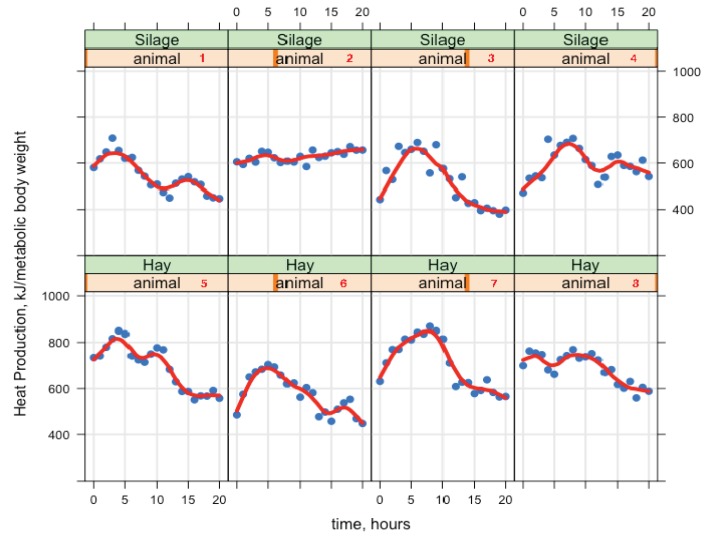
Heat production in 8 lactating dairy goats consuming diets composed of 40% forage (either alfalfa hay [Hay] or alfalfa silage [Silage]) and 60% of concentrate; observation data (points) and smooth regression (solid line).

**Figure 4:**
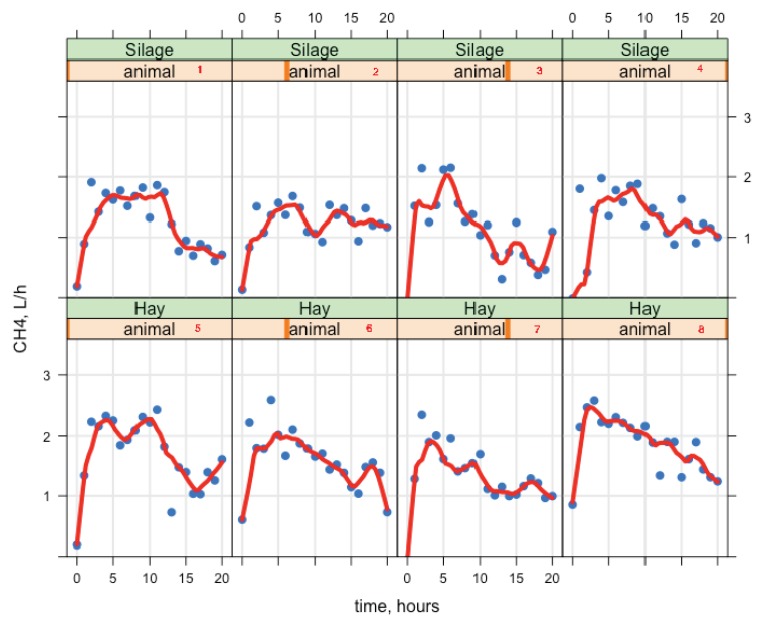
Methane (CH_4_) production in 8 lactating dairy goats consuming diets composed of 40% forage (either alfalfa hay [Hay] or alfalfa silage [Silage]) and 60% of concentrate; observation data (points) and smooth regression (solid line).

Goats were fed once per day and HP and CH_4_ changed over time. The effect of time was significant for gas exchange and it is not shown in the tables, but it is shown in Figure 3 and Figure 4 (postprandial profiles of the rates of HP and CH_4_ production (y-axis), x-axis represents the time of day). As gas exchange can increase and/or decrease over time throughout the day, when adding higher-order terms, the linear regression was able to reach the right model, but the observed data was not symmetrical. The HP showed a non-linear pattern, and after the feed intake a subsequent CH_4_ maximum was found. Therefore, a smoother locally weighted regression (loess; [24]) was also shown in Figure 3 and Figure 4; loess is a function for drawing a smooth curve with no assumptions about the form of the relationship. However, the average HP value was similar between diets (685 kJ/kg BW^0.75^ day; Table 6). By visualizing the pattern of HP over a day, we can observe that the greater intake was associated with greater HP and higher CH_4_ emissions (Figure 3 and Figure 4), and therefore, it is one positive aspect of the continuous gas exchange recording every two minutes with the open-circuit indirect calorimetry system.

There is little information on the variation of enteric CH_4_ emissions from dairy goats with different capacities for energy partitioning, and this may be attributed to the lack of energy metabolism data measured by indirect calorimeters. The daily rate in HP and CH_4_ from Figure 3 and Figure 4 shown that just after feeding HP and CH_4_ showed an immediate increase, which reflected eating activity and CH_4_ production. Greater HP and CH_4_ for HAY than for SIL diet was observed. Different authors [1] confirmed that dry matter intake (DMI) is the most important variable to predict enteric CH_4_ emission, and the significant positive relationship between DMI and CH_4_ emission demonstrates that as ruminants consume more feed, more CH_4_ is produced due to the greater availability of substrate for microbial fermentation. 

The data shown in Figure 4 argue against those approaches that attempt to estimate average daily CH_4_ production from measures lasting for only one hour or less. Although the lag time between a feed intake event (once a day in our trial) and the peak in postprandial CH_4_ production was relatively constant for dairy goats fed a mixed ration in our study, it varied greatly among individuals. Practical measurement of feed intake and CH_4_ in a grazing environment is a serious challenge, and the cost of measurement is an important issue. Therefore, other authors [18,19] have suggested that measuring the CH_4_ of grazing sheep for 40 to 60 min in portable accumulation chambers would also provide useful information on feed intake and efficiency; indeed, it has been proposed that CH_4_ measurements be included in selection indices.

Feed intake, milk yield and CH_4_ production are shown in Table 5. Differences of 0.47 kg DM intake/day were found between HAY and SIL diets, but the differences in DMI did not lead to significant changes in milk yield (1.59 kg/goat and day, on average). Goats fed the SIL diet produced lower (*p* < 0.05) CH_4_ emissions than those fed the HAY diet. The reduction of 5.1 g CH_4_ /day and goat in the SIL diet compared with the HAY diet suggests a DMI effect, because when CH_4_ emission was expressed per kg of DMI or per kg of milk yield, no differences were observed (16.4 g/kg DMI and 15.1 g/kg milk, on average). Therefore, a decrease in DMI of 0.47 kg/day reduced the CH_4_ emission in 5.4 g/day. The ratio CH_4_ to CO_2_ describes the proportion of the C excreted as CH_4_ (microbial fermentation of the feed) that is not metabolized to CO_2_. We found no significant differences in the ratio CH_4_ to CO_2_ between diets (0.065 on average). 

In ruminants, decreased production of CH_4_ can represent an improvement in feed efficiency, as ruminants lose between 2–12% of the gross dietary energy intake in the form of CH_4_ [25]. No differences between the two diets were observed for Ym (5.3% on average), and the value was in the middle of the range proposed by Johnson and Johnson [25], possibly due to feeding goats with mixed diets. The lack of differences between diets was attributed to the fact that diets were composed of similar feeds (hay or silage alfalfa and concentrates) and that the greater DMI was accompanied with greater CH_4_ emission. 

The results of the daily energy balance and HP determined by the RQ method are shown in Table 6. Gross energy intake, CH_4_ energy and MEI were larger for HAY than for SIL diet (474, 16 and 324 kJ/kg BW^0.75^ day, respectively). No significant difference was found for HP between diets as we mentioned above, with an average value of 685 kJ/kg BW^0.75^ day. 

There is little information on the relationship between CH_4_ emission and energetic efficiency of lactating dairy goats. We observed that Ym increased (from 5.0 to 5.6%) when increasing the inefficiency of ME use for production (HP/MEI); the inefficiency was 61% and 82% for the HAY and SIL diets, respectively. Other research [26] carried out with Alpine goats consuming 60% concentrate diet at mid lactation (Ym = 4.9) obtained an inefficiency (HP/MEI) of 52%. Other authors [27] working with Saanen goats at mid lactation obtained a Ym of 4.7 and an inefficiency of 48%. Therefore, goats with high genetic merit had reduced CH_4_ emissions because they were more efficient in the use of the energy of the diet.

Due this, Ym was reduced when increasing the proportion of MEI; more ME was directed to milk production and body tissues (E_milk_ corrected/MEI). In our study, when Ym changed from 5.6 to 5.0%, the efficiency increased from 23% to 39% (E_milk_ corrected/MEI). In the study with Alpine goats [26], this efficiency was 47%, and in the study of Bava et al. [27], the efficiency was 51%, again being more efficient than the Murciano-Granadina goats used in our study. These comparisons demonstrate that dairy goats with high genetic merit had greater energy efficiency and produced less CH_4_. It seems that high genetic merit dairy goats are capable of partitioning more energy into milk than low genetic merit goats, as previously demonstrated [28] in dairy cows.

The daily CN balances are displayed in Table 7. Significant differences were found for C intake (43.4 vs. 33.2 g/kg BW^0.75^ day for HAY and SIL diets, respectively), and N intake (3.2 vs. 2.6 g/kg BW^0.75^ day for HAY and SIL diets, respectively). No significant differences in HP were found between diets (668 kJ/kg BW^0.75^ day) with the CN method, and values were lower than those obtained with the RQ method as we discussed above.

The efficiency of milk C output regarding C ingested was 22% and 24% for HAY and SIL diets, respectively. Therefore, energetically, goats fed these diets metabolize more feed carbon to milk production than goats fed mixed diets with alfalfa hay as forage from the study of Just et al. [29]; 16%. However, the ratio between milk N output and N ingested was similar between diets and with the study of Fernández et al. [30]; 22%.

Consequently, continuous measurements of O_2_, CO_2_ and CH_4_ concentration with the open-circuit indirect calorimetry system was suitable for HP determinations and CH_4_ quantifications in small ruminants. As described by Derno et al. [31], indirect calorimetry, together with on-line measurements of feed intake and sensors that determine standing and lying positions, is becoming an indispensable tool to incorporate to our system in the future. 

## 4. Conclusions

Open-circuit indirect calorimetry system based on the head hood described here is a useful tool for continuously monitoring various components of energy metabolism and GHG. It also allows dynamic changes in these variables to be recorded over the course of a day or for longer periods. We expect this will help research to understand the complex mechanism of energy metabolism and relationship between diet and CH_4_ emission. Some advantages of the system are that it allows animals to eat, drink and lay down, CH_4_ recovery is over 99% accurate and it is cheaper that respiration chambers. Disadvantages are that animals have to be trained, animal behavior could be compromised, reducing the intake, and it is not applicable for grazing animals. The data acquisition and recording device developed improved the accuracy of the indirect calorimetry system by reducing the work involved in managing output data and refining the functionality for measuring gas exchange and energy metabolism in small ruminants.

## Figures and Tables

**Table 1:** Ingredients and chemical composition of the diets.

Ingredients and Chemical Composition	Diet ^1^	
Item	HAY	SIL
Ingredients, g/kg DM		
Alfalfa Hay	400	
Alfalfa Silage		400
Barley	210	210
Corn	185	185
Wheat bran	90	90
Soy meal (44% Crude Protein)	89	89
Calcium carbonate	13	13
Sodium chloride	7	7
Bypass fat ^2^	3	3
Premix ^3^	3	3
Chemical composition, % of DM		
Dry matter	91.3	90.8
Organic matter	88.5	87.6
Crude Protein	19.0	20.1
Ether extract	2.0	2.9
Neutral detergent fiber	29.9	28.1
Acid detergent fiber	16.0	16.8
Acid detergent lignin	3.1	3.0
Starch	17.3	17.3
Carbon	40.1	40.7
Nitrogen	2.9	3.2
Gross energy ^4^, MJ/kg DM	16	17

^1^ Diets were composed of 40% forage (either alfalfa hay [HAY] or alfalfa silage [SIL]) and 60% of concentrate. ^2^ Bypass fat of palm fatty acid distillate. Provided by Norel Animal Nutrition, Norel S.A., Spain.^3^ Provided by NACOOP S.A. España. Premix composition (ppm or UI per kilogram of premix): Se, 40; I, 250; Co, 80; Cu, 3000; Fe, 6000; Zn, 23400; Mn, 29,000; S, 60,000; Mg, 60,000; vitamin A, 2,000,000 UI; vitamin D3, 400,000; vitamin E, 2000 ppm; nicotinic acid, 10,000; choline, 20,300.^4^ DM = dry matter.

**Table 2:** Calibration factors (mean and standard deviation; S.D.) of the indirect calorimetry system (n = 6).

Calibration Gases	Head Hood 1		Head Hood 2	
	mean	S.D.	mean	S.D.
O_2_	1.001	0.0007	1.009	0.0006
CO_2_	1.025	0.0012	1.002	0.0011
CH_4_	0.986	0.0031	0.989	0.0039

**Table 3:** Repeatability study of the open-circuit indirect calorimetry system.

Variation Measurement	CH_4_		HP ^1^	
	VarComp ^2^	Contribution (%)	VarComp ^2^	Contribution (%)
Total Gage R&R	0.29	79	9965	61
Repeatability	0.29	79	9965	61
Part to part	0.07	21	6119	39
Total variation	0.36	100	16084	100

^1^ HP = heat production. ^2^ VarComp = variance component.

**Table 4:** Heat production (kJ/kg BW^0.75^ day) of 8 lactating Murciano-Granadina goats measured by the Respiration Quotient (RQ)and Carbon Nitrogen (CN) methods.

MEI ^1^	HP ^2^; RQ Method	HP; CN Method	Discrepancy ^3^
1015	639	605	3.31
1074	631	616	1.48
903	609	612	−0.33
1378	733	725	0.59
840	832	773	7.05
786	605	584	2.57
897	781	773	0.89
980	649	651	−0.18
		mean	1.92

^1.^ MEI = metabolizable energy intake. ^2^ HP = heat production. ^3^ discrepancies between RQ and CN methods = [(HP_RQ_ − HP_CN_)/MEI] × 100.

**Table 5:** Intake (DMI), milk yield, CH_4_ emission, breath CH_4_/CO_2_ ratio, and CH_4_ yield (g/kg) of 8 lactating Murciano-Granadina goats fed HAY (n = 4) or SIL (n = 4) diets.

Item ^1^	Diet ^2^		S.E.M. ^3^	*p*-Value
	HAY	SIL		
body weight, kg	39.9	37.9	2.24	0.675
total DMI, kg/d	1.72	1.25	0.103	0.0095
Milk yield, kg/goat	1.67	1.51	0.084	0.5452
CH_4_, g/d	26.6	21.5	1.69	0.048
CH_4_/CO_2_ ratio	0.07	0.06	0.005	0.9873
Ym, %	5.0	5.6	0.28	0.824
CH_4_/DMI, g/kg	15.5	17.3	0.90	0.720
CH_4_/milk, g/kg	15.9	14.3	0.80	0.681

^1^ DMI = dry matter intake; Ym = methane energy divide by gross energy intake. ^2^ Diets were composed of 40% forage (either alfalfa hay [HAY] or alfalfa silage [SIL]) and 60% of concentrate. ^3^ S.E.M. = standard error of the mean.

**Table 6:** RQ method; daily energy balance (kJ/kg BW^0.75^ day) of 8 lactating Murciano-Granadina goats fed HAY (n = 4) or SIL (n = 4) diets.

Item ^1^	Diet ^2^		S.E.M. ^3^	*p*-Value
	HAY	SIL		
Intake, g/kg BW^0.75^	110.1	81.8	6.33	0.013
GEI	1866	1392	107.2	0.015
E_feces_	586	461	34.9	0.069
E_urine_	41	32	4,7	0.363
E_methane_	93	77	6.0	0.048
MEI	1146	822	75.3	0.011
E_milk_	477	403	38.3	0.364
O_2_, L/h	22.0	20.7	1.10	0.572
CO_2_, L/h	20.9	19.5	1.04	0.530
CH_4_, L/h	1.54	1.25	0.091	0.0481
RQ	0.95	0.94	0.014	0.809
HP_RQ_	699	671	36.0	0.737
RE_body_	-30	-252	69.2	0.075

^1^ GEI = gross energy intake; E_feces_ = energy losses in feces; E_urine_ = energy losses in urine; E_methane_ = energy losses in methane; MEI = metabolizable energy intake; E_milk_ = energy in milk; RQ = respiration quotient; HP_RQ_ = heat production according to RQ method; RE_milk_ = recovered energy in milk; RE_body_ = recovered energy in tissue (REbody = MEI − HP − Emilk); kl = efficiency of use of ME for milk production. ^2^ Diets were composed of 40% forage (either alfalfa hay [HAY] or alfalfa silage [SIL]) and 60% of concentrate. ^3^ S.E.M. = standard error of the mean.

**Table 7:** CN method; carbon and nitrogen balance (g/kg BW^0.75^ day) of 8 lactating Murciano-Granadina goats fed HAY (n = 4) or SIL (n = 4) diets. RE and HP are expressed in (kJ/kg BW^0.75^ day).

Item ^1^	Diet ^2^		S.E.M. ^3^	*p*-Value
	HAY	SIL		
C_intake_	43.4	33.2	2.41	0.023
C_feces_	14.2	11.2	0.85	0.073
C_urine_	1.0	0.8	0.12	0.449
C_CO2_	17.2	16.4	0.92	0.694
C_CH4_	1.3	1.1	0.08	0.772
C_milk_	9.5	8.0	0.76	0.361
C_retained body_	0.2	−4.3	1.37	0.077
N_intake_	3.2	2.6	0.15	0.047
N_feces_	1.0	0.8	0.06	0.073
N_urine_	0.6	0.5	0.08	0.790
N_milk_	0.7	0.6	0.06	0.279
N_retained body_	0.9	0.7	0.12	0.448
RE _body_	−7	−238	69.47	0.074
HP_CN_	639	658	35.97	0.753

^1^ C_intake_ = C intake; C_feces_ = C losses in feces; C_urine_ = C losses in urine; C_CO2_ = C losses in CO_2_; C_CH4_ = C losses in methane; C_milk_ = recovered C in milk; C_retained body_ = recovered C in tissue; N_intake_ = N intake; N_feces_ = N losses in feces; N_urine_ = N losses in urine; N_milk_ = recovered N in milk; N_retained body_ = recovered N in tissue; RE_body_ = recovered energy in tissue; HP_CN_ = heat production according to CN method. ^2^ Diets were composed of 40% forage (either alfalfa hay [HAY] or alfalfa silage [SIL]) and 60% of concentrate. ^3^ S.E.M. = standard error of the mean.
